# Molecular characterization of *Trypanosoma evansi*, *T. vivax* and *T. congolense* in camels (*Camelus dromedarius*) of KSA

**DOI:** 10.1186/s12917-022-03148-0

**Published:** 2022-01-18

**Authors:** Jamila S. Al Malki, Nahed Ahmed Hussien

**Affiliations:** Department of Biology, College of Science, Taif University, P.O. Box 11099, Taif, 21944 Saudi Arabia

**Keywords:** Trypanosomosis, ITS1, Rotat 1.2 VSG, Phylogeny, Taif governorate, KSA

## Abstract

**Background:**

*Trypanosoma evansi* is the leading infectious *Trypanosoma* spp*.* in camels (*Camelus dromedarius*) present in the Kingdom of Saudi Arabia (KSA) that could lead to extensive economic losses. The present study was aimed to assess the prevalence rate of *T. evansi* in Taif governorate, Makkah province, KSA using parasitological and molecular evaluations, and analyze their genetic relationship targeting internal transcribed spacer 1 (ITS1) and variable surface glycoprotein (VSG) genes. For evaluation, we have used 102 blood samples of camels obtained from three different regions in Taif.

**Results:**

Results show a considerable prevalence rate of trypanosomosis 2/102 (2.0%) according to Giemsa-stained buffy coat smear, and 16/102 (15.7%) according to touchdown PCR. *T. evansi* (*n* = 10/102, 9.8%) was the main infectious species found in camels then *T. vivax* (*n* = 3/102, 2.9%). Mixed infections were detected in three camels with *T. evansi*, *T. vivax*, and *T. congolense* (*n* = 3/102, 2.9%). Regarding gender, the results indicate that female camels (11/66, 16.7%) show higher prevalence of *Trypanosoma* than males (5/36, 13.9%). Sequencing and phylogenetic analyses of ITS1 and VSG showed their relationships with *T. evansi* in other hosts from different countries.

**Conclusions:**

In our peer knowledge, it is the first time to report a research-based prevalence of trypanosomosis in the camels of Taif governorate, Makkah province, KSA.

**Supplementary Information:**

The online version contains supplementary material available at 10.1186/s12917-022-03148-0.

## Background

Saudi Arabian camels are mainly of the type one-humped (dromedaries, *Camelus dromedarius*), representing a significant and integral component of the Kingdom heritage. According to the FAO 2019, the Kingdom of Saudi Arabia (KSA) has about 34% (492,853) of the total population of dromedary present in the Arabian Peninsula (estimated 1.46 million) [1]. Camel population in KSA represents about 51% of the total tropical livestock unit that increased since 1961 (first available world annual data) [2]. Camels have particular importance in KSA; they are reared for their milk, wool, leather, and meat, in addition to their contribution in racing and cultural festivals, but rarely used for transportation [3].

*Trypanosoma evansi* (*T. evansi*) is a protozoan parasite that infects different animals, including horses, donkeys, and dogs [4, 5], camels, sheep, and goats [6], and a potential human pathogen. It causes trypanosomosis that represents a significant threat to an animal’s life [7, 8]. *Trypanosoma evansi* has a complex and multiple means of transmission that depends on animal host species, biting/sucking insects, geographical area, and mode of transmission, leading to its epidemiological significance in different parts of the world [9].

Camel represents the main host of *T. evansi* in Middle Asia and certain parts of Africa such as West Africa, Sudan, Kenya, and Somalia [10]. Trypanosomosis is present in acute and chronic forms, with different symptoms including fever, anemia, edema, bodyweight loss, lacrimation, conjunctival petechiae, abortion, enlarged lymph nodes, decreased fertility, and could lead to death [11].

There is a scarcity of studies reporting the prevalence of trypanosomosis in different KSA governorates. However, Al-Qassim and Riyadh provinces are the most interesting regions for studying trypanosomosis in KSA [12]. Few studies suggested that tick *Hyalomma dromedarii* and tabanid flies could be vectors for camel trypanosomosis in KSA. However, they have found *Trypanosoma* developmental stages in *Hyalomma dromedarii* salivary glands during their examination. Moreover, they have observed many tabanid flies feeding on camels in the regions where camels showed *T. evansi* infection, a mechanical vector [13, 14].

The present study evaluated the prevalence of camel trypanosomosis, especially *T. evansi*, in Taif governorate, Makkah province, KSA using parasitological and molecular methods. In addition, the present study assessed the genetic relationship between internal transcribed spacer 1 (ITS-1) and RoTat 1.2 variable surface glycoprotein (VSG) Taif isolates with other isolates present in other countries in Genbank.

## Results

### Buffy coat examination

**Fig. 1:**
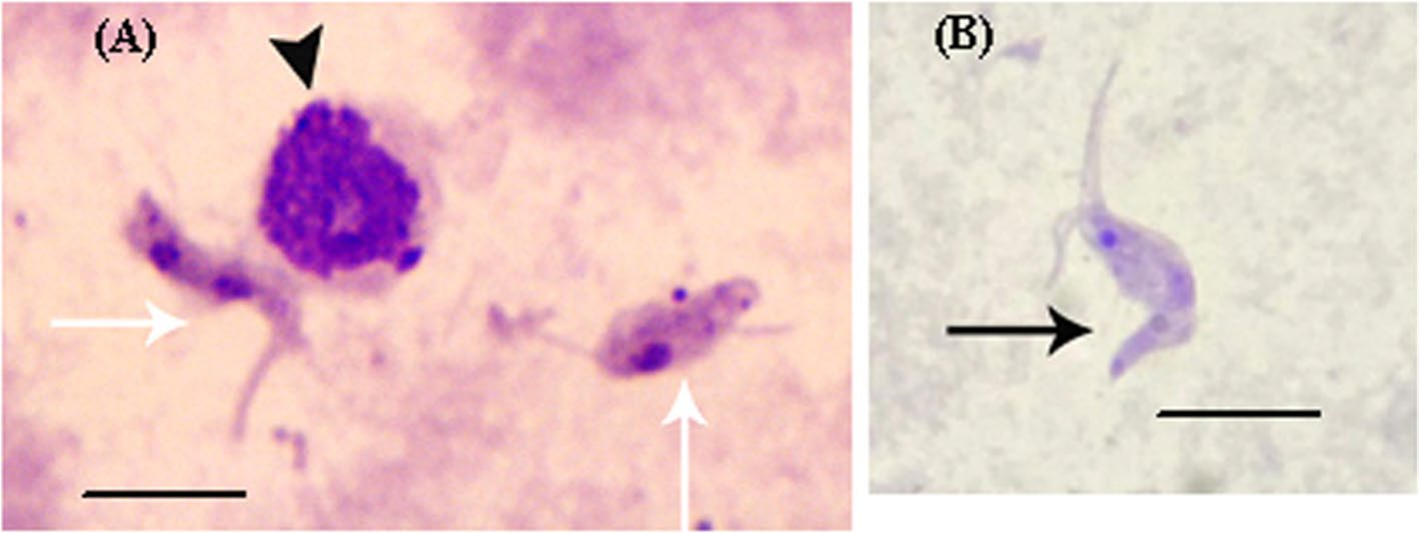
Light micrographs of camels’ blood buffy coat smear showing Giemsa-stained *Trypanosoma evansi* with long terminal free flagella (white (**A**) and black arrows (**B**)) and apoptotic lymphocyte (black arrowhead, (**A**)). Scale-bars: 10 μm

Parasitological examination, using Giemsa stain, in the whole blood smear of all samples (*n* = 102) showed negative results. However, Giemsa staining of buffy coat smear showed positive *T. evansi* with long terminal free flagella in only two samples (1 male and 1 female, 2.0%) (Fig. 1, Table 1). Most of them appeared deformed/lysed in the same two samples (Data not shown). In addition, Fig. 1 showed apoptosis of white blood cell due to trypanosomosis.

**Table 1:** Prevalence of trypanosomosis according to parasitological and molecular assays

Gender	Number (***n***)/Age	Buffy coat parasitological detection	PCR detection	***Trypanosoma*** spp. detected by PCR detected
**Female**	*n* = 66 Age (3–12 yrs)	1/66 (1.5%)	11/66 (16.7%)***	*T. evansi* = 10/102 (9.8%)*** *T. vivax* = 3/102 (2.9%) ^a^Mixed =3/102 (2.9%)
**Male**	*n* = 36 Age (1–7 yrs)	1/36 (2.8%)	5/36 (13.9%)	
**Total**	102	2/102 (2.0%)	16/102 (15.7%)	16/102 (15.7%)

Detection of *Trypanosoma* spp. in blood of camels

In which, *** indicates *P* ≤ 0.001 comparing related samples with each other

^a^Mixed infection of *T. evansi* and *vivax* was detected by Kin primers, while mixed infection of *T. evansi*, *vivax*, and *congolense* was detected by ITS1 primers

### Touchdown PCR

PCR was done to detect *Trypanosoma* spp*.* by targeting internal transcribed spacer (ITS) of nuclear ribosomal DNA (rDNA), ITS1, and RoTat 1.2 variable surface glycoprotein (VSG). Touchdown PCR is a good solution for us because we failed to obtain a good amplicon product using conventional PCR with one fixed annealing temperature.

**Fig. 2:**
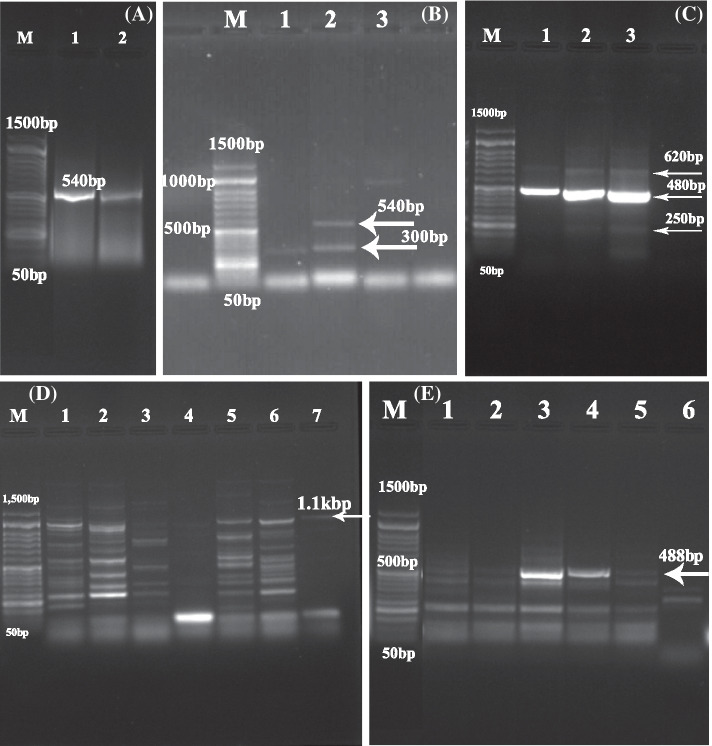
Agarose gel stained with ethidium bromide (1.5%) showing PCR product of ITS1 using Kin primers (**A** and **B**). Gel (**A**) Lanes 1,2: *Trypanosoma evansi* (540 bp), Gel (**B**) Lane 1: *T. vivax* (300 bp), and Lane 2: Mixed infection of *T. evansi* and *T. vivax*. Gel (**C**) showing PCR product of ITS1 using ITS1 primers; Lanes 1, 2, 3: Mixed infection of *Trypanosoma evansi* (480 bp), *T. vivax* (250 bp), and *T. congolense* (620 bp). Gel (**D**) showing PCR product of ITS using IR primers; Lanes 1–7: *Trypanosoma evansi* (1.1 kbp). Gel (**E**) showing PCR product of Rotat 1.2 VSG region using ILO primers; Lanes 1–6: *Trypanosoma evansi* (488 bp). Any other lower and higher bands are non-specific. Lane M: Low molecular weight marker (50–1500 bp)

PCR results revealed infection of 16 camels (15.7%) with *Trypanosoma* spp. Females have a significant infection rate higher (16.7%) than males (13.9%) at *P* ≤ 0.001 (Table 1). PCR results using Kin primers that targeted ITS1 showed 16 positive samples, in which 10 samples have PCR product at 540 bp, 3 samples with 300 bp, and 3 samples mixed between 540 bp and 300 bp that referred to *T. evansi*, *T. vivax*, and mixed infection of both *T. evansi* and *T. vivax*, respectively (Fig. 2A, B).

Using ITS primers that targeted ITS1 region reported ten samples with *T. evansi* infection (480 bp) and three mixed infection samples of *T. evansi* (480 bp), *T. vivax* (250 bp), and *T. congolense* (620 bp) (Fig. 2C). At the same time, IR primers were used to amplify ITS region (ITS1 + 5.8S + ITS2 rDNA) of *T. evansi* that detected ten samples with a main 1.1 kbp band and other non-specific bands (Fig. 2D). Rotat 1.2 VSG region for *T. evansi* was detected in 10 samples using ILO primers with amplicon size 488 bp, while other lower and higher bands are non-specific (Fig. 2E). Therefore, *T. evansi* (*n* = 10/102, 9.8%) has been significantly reported at *P* ≤ 0.001 by PCR in comparison to *T. vivax* (*n* = 3/102, 2.9%), and then mixed infections *T. evansi, T. vivax,* and *T. congolense* (*n* = 3/102, 2.9%) as mentioned in Table 1.

### Sequencing and phylogeny

Since *T. evansi* is the most common infectious species in Taif camels than *T. vivax* and T. *congolense*, we randomly selected about twelve different *T. evansi* PCR products samples using Kin (540 bp), ITS (480 bp), and ILO (488 bp) primers and were sequenced. It is hard to sequence the PCR product of IR primers (1.1 kbp) because it contains other non-specific bands.

**Fig. 3:**
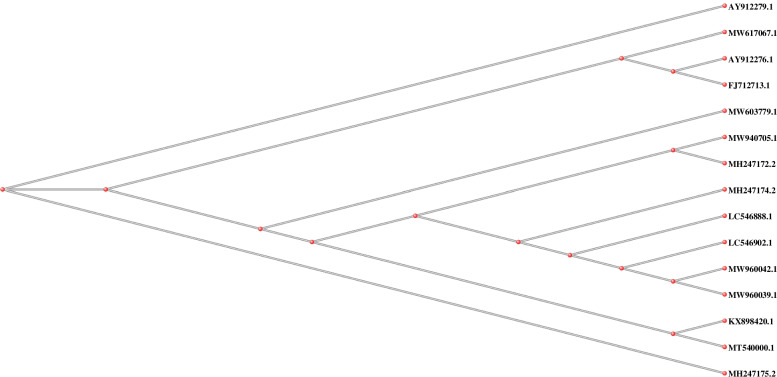
Phylogenetic relationships between Taif_camel *T. evansi* (ITS1) MW960042, and MW960039 (using Kin primers), and MW940705 (using ITS primers) with other reference sequences of *T. evansi* from NCBI GenBank. *Trypanosoma* GenBank sequences were shown by their accession numbers, country, and host

**Fig. 4:**
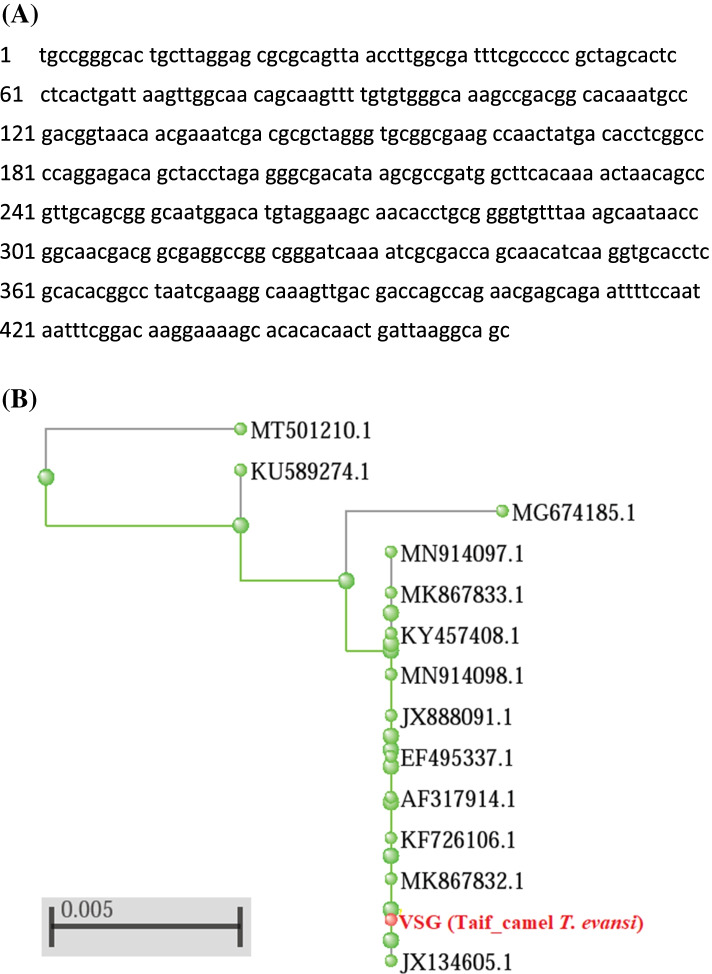
FASTA sequence of Rotat 1.2 VSG region using ILO primers (**A**). Its relationship with other reference sequences of *T. evansi* from NCBI GenBank using phylogenetic tree (**B**). *Trypanosoma* GenBank sequences were shown by their names, host names (if present in Genbank), and accession numbers. Bar scale represents 0.005 nucleotide substitution per site

Selected sequences were deposited in Genbank and have assigned different accession numbers: MW940705 for ITS1 (one sample using ITS primers), MW960042, and MW960039 for ITS1 (two samples using Kin primers), and RoTat 1.2 VSG (two samples using ILO primers, their sequences are present as supplementary files, unpublished yet) regions, respectively. MW940705, MW960042, MW960039, and VSG were blasted with various related *Trypanosoma* spp*.,* especially for *T. evansi* in camels’ blood sequences from other different countries (e.g., Kenya, Sudan, Somalia, China, Iran, Egypt, etc.). Phylogenetic trees were constructed from these sequences, as shown in Figs. 3 and 4.

Alignment of MW960042 with MW960039 showed identity with 97%. Likewise, MW960042 showed 98% alignment identity with *T. evansi* LC546902.1 (Philippines), MW617067 (Egypt), FJ712713 (China), AY912276 (Thailand), MT540000 (South Algeria), MH247175 (Kenya), and MH247174 (Kenya).

MW940705 (ITS1 region using ITS primers) showed 100% alignment identity with *T. evansi* ITS1 MT225591.1 (from India), MH247175.2 (Kenya), MH247174.2 (Kenya), MH247172.2 (Kenya), and LC546888.1 (Philippines), 99.77% alignment with KX898420.1 (from Iran), MW603779.1 (Egypt), AY912279 (Thailand), and MT540000 (Algeria). Figure 3 shows MW960042, MW960039, and MW940705 molecular relationship with other most related ITS1 *T. evansi* from different countries in various hosts as a slanted cladogram.

However, RoTat 1.2 VSG gene sequence of Taif_camel blood samples revealed 100% alignment identity with *T. evansi* VSG MK867833.1 (from Kenya), JX134605.1 (India), and KU589274.1 (India), 99% with JX888091 (Egypt), and 98% with LC493167 (Sudan) according to NCBI BLAST. Sequence relationship with other most related neighbored sequences was constructed in rectangular cladogram as shown in Fig. 4.

## Discussion

In the present study, we have evaluated trypanosomosis, especially *T. evansi,* prevalence in camels (*n* = 102) found in Taif governorate in Makkah province of KSA. It is easy to inventory the number of camels in the present study region because most camels were officially numbered in KSA according to the camel’s electronic numbering project according to MEWA. It was not easy to find any *Trypanosoma spp.* in the blood smears of camels. Accordingly, for a more specific and sensitive microscopic parasitic diagnosis, we have used Giemsa-stained buffy coat smear, rather than using the whole blood method, which is used as well for *Haemoproteus, Leucocytozoon*, *Plasmodium*, filarioid helminths, *Trypanosoma*, and *Lankesterella parasites* examination [15]. Unfortunately, only two samples, 2% (1 male and one female), have positive results of *T. evansi* infection, and most detected parasites are deformed.

A high prevalence of *Trypanosoma* spp*.* (15.7%) in camels of Taif governorate was reported using PCR, where prevalence among females was significantly higher than that of males (16.7 and 13.9%, respectively). Also, infection caused by *T. evansi* was significantly higher than caused by *T. vivax* (9.8 and 2.9%, respectively). However, prevalence of mixed infection caused by *T. evansi*, *T. vivax*, and *T. congolense* together was 2.9%. Low parasitemia, typical for the chronic infection phase, explains the difference of positive results between parasitological and molecular assessment [16]. Further studies have revealed that *T. evansi* is the first leading cause of trypanosomosis in camels, followed by *T. vivax*, *T. congolense*, *T. brucei*, and *T. simiae* [17]. *T. evansi* affects many wild and domestic mammals in South America, Asia, and Africa. Globally, *T. evansi* has its highest prevalence rate in camels than other animal hosts such as horses, dogs, buffaloes, and cattle. Nearly all biting flies could transmit this species as mechanical vectors; therefore, they have potentially unlimited geographical reach [18].

In our peer knowledge, it is the first time to report the prevalence of trypanosomosis in Taif governorate of Makkah province. The present study showed a lower prevalence rate of *Trypanosoma* spp*.* (15.7%), especially *T. evansi* (9.8%), in this region than other regions of Saudi Arabia that have been previously studied. In which, Metwally et al. [12] reported a high prevalence rate of *T. evansi* in Al-Qassim (46%) and Riyadh (39.5%) provinces according to molecular evaluation targeting ITS1 gene. We have reported a significantly high prevalence according to sex in females than males, which is consistent with Metwally et al. [12] and inconsistent with other studies in Saudi Arabia and Iraq [13, 19]. They suggested that the high infection rate in female camels may be returned to low management, traveling through a high vector burden area, and favoritism by biting insects [20]. Considering the number of females included in the present study is more than the number of males because males were slaughtered than females, that gives a chance to spread infection within females.

On the other hand, Al-Afaleq et al. [10] have reported a low prevalence of patent trypanosomosis from the western to the southern regions of KSA (ranges from 0.6–2%) according to buffy coat parasitological examination. However, the prevalence rate increased based on serological evaluation by using CATT/*T. evansi* (39.4%). *Trypanosoma* prevalence varied from region to other in KSA and was consistent with other studies in different countries. In Iran, the prevalence rate of *T. evansi* infection has been reported in dromedary camels varied between zero to 19.47% in various regions [21].

The lower prevalence rate of trypanosomosis, especially *T. evansi*, in camels of Taif governorate than others in KSA could be returned to various factors. According to the ministerial recommendation, a periodic follow-up examination of camels and wide use of anti-trypanosomal drugs of those reared camels could be the main factor. Anti-trypanosomal drugs have been approved and used in Saudi Arabia, such as Trypomidium-Samorin (isometamedium cholride, Merial), Triquin (quinapyramine, Wock-herde), and Cymelarsan (melarsomine, Merial) [22], that have shown their efficiency in KSA and other neighboring countries, such as the United Arab Emirates [23]. The change of the usage of trypanocides frequency might have a role in prevalence diversity in different regions of KSA [9]. In addition, the abundance of pathogen vectors such as ticks was affected by altitude, temperature, humidity, and saturation deficit. Therefore, high altitude region of Taif and moderate climate could have a role in the low abundance of ticks as previously referred [24]. Gilbert [24] suggested that tick abundance would be higher at lower altitudes, warmer climates that could have potential pathogen prevalence implications.

We have targeted ITS (using IR primers), especially ITS1 (using ITS and Kin primers), and VSG (using ILO primers) regions for molecular determination of *T. evansi* because they are reliable detection targets [25–27] and are used for delineation of species and phylogenetic relations of *Trypanosoma* spp*.* [28, 29]. Isolates of ITS1 and VSG have shown phylogenic relationships with other isolates of *T. evansi* in different hosts of the Philippines, Egypt, China, Thailand, Kenya, Iran, Sudan, and other countries, as reported in Metwally et al. [12]. However, sequencing more isolates for phylogenic analysis could be useful for the relationship assessment of *T. evansi* in the blood of camels found in Taif governorate, Makkah province, KSA. Finally, there is a need to establish several control policies to decrease trypanosomosis in KSA. Camels’ vaccination, control of ticks and pathogen-borne vectors, consistent examination, and import from authorized countries that their animals were free from any pathogens could help control infection rates.

## Conclusions

Results report a considerable prevalence rate of trypanosomosis, especially *T. evansi*, in the blood of camels reared in the high-altitude region, Taif governorate, of Makkah province in KSA using parasitological and molecular evaluation. However, this prevalence rate is lower than different other governorates in KSA. This reflects health care awareness towards reared camels, follow-up examinations, and medical treatment, if needed, according to ministerial recommendations. In addition, there is a need to establish measures and stringent control policies to help prevent the parasite spread. We also recommend updating the disease prevalence data throughout the whole kingdom that could share in changing the infection situation. In addition, it is necessary to expand phylogenetic analyses for *T. evansi* identification with other more taxa with other molecular markers.

## Methods

### Study area

**Fig. 5:**
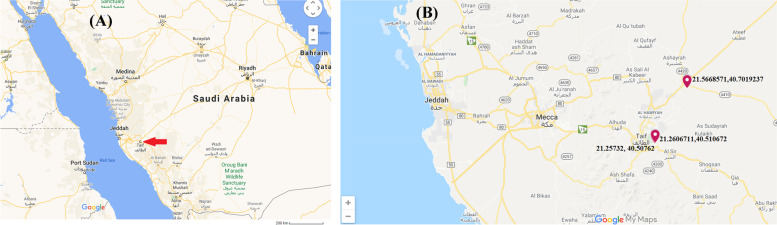
Map of Saudi Arabia showing Taif governorate (**A**, red arrow), (**B**) referring to the latitude and longitude coordinates of the present study area of collecting samples according to Google map 2021 (Map source: adapted from Google Maps® 2021)

Taif governorate is a high-altitude region (about 1.87 m above sea level) in Makkah province of KSA with a special climate due to its geographical position and altitude. According to Köppen and Geiger, the Taif climate is classified as a hot desert climate (BWh) [30]. We have selected the most popular areas in Taif that found camels grazed in to collect our data/samples. Most of the camels in Taif governorate were found grazing in three different regions with latitude and longitude coordinates 21°5,668,571 N 40°7019237E, 21°25,732 N 40°50762E, and 21°2,606,711 N 40°510672E, respectively (Fig. 5). It is easy for us to locate and collect their data because most camels were officially numbered according to the camel’s electronic numbering project established in KSA as an obligatory measure since 2017 according to the Ministry of Environment, Water, and Agriculture (MEWA).

### Animals’ data and sample collection

According to the ministerial recommendation, camels (*Camelus dromedarius*) were subjected to periodic examination by veterinarians. Blood samples were collected from their side to ensure that animals were free from diseases. Data about camels such as age, gender, other different data, and 2 ml of collected blood samples (from ear veins) in EDTA tubes were introduced from veterinarians as per their periodic examination. Data and blood samples were collected from the period December 2020 to February 2021. At the time of blood collection, camels were clinically healthy. A total of 102 samples, 66 females (age ranges from 3–12 years) and 36 males (age ranges from 1–7 years), were evaluated.

### Parasitological evaluation

Blood and buffy smears were done for all samples, stained with 10% Giemsa, and examined under a light microscope for *Trypanosoma* spp*.* diagnosis [31].

### DNA extraction

DNA was extracted manually by salting out. First, a 0.5 ml blood sample was mixed with the same volume of low salt buffer TKM1 (100 mM Tris-HC1, pH 7.4, 250 mM sucrose, 10 mM EDTA) and incubated at room temperature until complete blood cells’ lysis. Next, the mixture was centrifuged (4000 rpm, 10 mins), and then 480 μl of high salt buffer TKM2 (Tris HCl 10 mM pH 7.6, 10 mM KCl, 10 mM MgCl_2_, 0.4 M NaCl, and 2 mM EDTA), 75 μl 10% SDS, and 10 μl proteinase k enzyme (10 mg/ml) were added to the pellet and incubated at 55 °C for 30 mins. Protein was precipitated by the aid of 6 M NaCl, centrifuged (11,500 rpm, 20 mins), and then supernatant was transferred to new eppendorf for DNA precipitation by cold ethanol addition. Next, the DNA pellet was collected by centrifugation (10,000 rpm, 10 mins), washed, dried, and finally dissolved in 100 μl autoclaved Milli-Q water [32].

### PCR amplification

The internal transcribed spacer (ITS) region (consists of ITS1 + 5.8S + ITS2 rDNA), ITS-1, and RoTat 1.2 variable surface glycoprotein (VSG) regions of *T. evansi* were targeted by different primers as mentioned in Table 2. For-Kin, Rev-Kin; For-ITS1, Rev-ITS1; and For-IR, Rev-IR primers were used for ITS1, and ITS different regions amplification, while For-ILO and Rev-ILO were used for RoTat 1.2 (VSG) region amplification as previously cited [33–36].

**Table 2:** Primers and target genes of *Trypanosoma* spp*.* investigated [33–36]

Primers (5′-3′)	Target gene	Product size (bp)	Cycling conditions (same for all primers)
**For-Kin:** GCGTTCAAAGATTGGGCAAT	ITS1	*T. evansi* (540 bp), *T. vivax* (300 bp)	94 °C—3 min 94 °C—1 min 58 °C—1 min (× 4) 72 °C—1 min 94 °C—1 min 56 °C—1 min (× 8) 72 °C—1 min 94 °C—1 min 54 °C—1 min (× 23) 72 °C—1 min 72 °C—5 min [37]
**Rev-Kin:** CGCCCGAAAGTTCACC			
**For-ILO:** GCCACCACGGCGAAAGAC	Rotat 1.2 VSG	*T. evansi* (488 bp)	
**Rev-ILO:** TAATCAGTGTGGTGTGC			
**For-IR:** GCTGTAGGTGAACTTGCAGCAGCTGGATCATT	ITS	*T. evansi* (1.1 kbp)	
**Rev-IR:** GCGGGTAGTCCTGCCAAACACTCAGGTCTG			
**For-ITS1:** CCGGAAGTTCACCGATATTG	ITS1	*T. evansi* (480 bp), *T. vivax* (250 bp), *T. congolense* (620 bp).	
**Rev-ITS1:** TTGCTGCGTTCTTCAACGAA			

PCR amplification was done in a total reaction volume of 20 μl: 7 μl H_2_O, 1 μl (20 pmole) of each forward and reverse primers, 1 μl extracted DNA, and finally 10 μl of 2× master mix. Touchdown PCR reaction was setup over 35 cycles as shown in Table 2 [37]. PCR products of all samples were loaded and separated at 2% ethidium bromide-stained agarose gel in a TBE buffer (1X) for about 45 min (100v), visualized, and then photographed by a gel documentation system. The size of PCR products was determined visually by comparing them with a known low molecular weight marker (50–1500 bp).

### Sequencing and phylogenetic assessment

Positive PCR products of *T. evansi* were selected and subjected to sequencing using an ABI Prism 3730 Genetic Analyzer automated sequencer. Sequences of different amplicons of ITS1 and RoTat 1.2 (VSG) regions were submitted in Genbank with the help of NCBI Genome Workbench software (version 3.6.0, built on 03/01/2021) to have accession numbers. Sequences of different regions of ITS, ITS1, and RoTat 1.2 (VSG) were aligned with various sequences in Genbank, estimated phylogenetically, and finally viewed as slanted/rectangular cladogram in the phylogenic Tree View by using NCBI Genome workbench and online NCBI BLAST [38].

### Statistical analysis

Statistical analysis was conducted to differentiate between different groups by using One-way ANOVA using GraphPad software (GraphPad® 2017, San Diego, CA, USA). *** indicates *P* ≤ 0.001, ** indicates *P* ≤ 0.01, * indicates *P* ≤ 0.05 and ns (non-significant) means *P* > 0.05.

## Supplementary Information

**Additional file 1.**

**Additional file 2.**

## Data Availability

The datasets analysed during the current study are available in Genbank with accession numbers MW940705, MW960042, and MW960039 for ITS1 region. VSG sequences are available as PDF supplementary files.

## Abbreviations

BWh: Hot deserts climate
ITS: Internal transcribed spacer
KSA: Kingdom of Saudi Arabia
MEWA: Ministry of Environment, Water, and Agriculture
*T. evansi*: *Trypanosoma evansi*
VSG: Variable surface glycoprotein
