# Supplementary material for: Molecular characterization of Trypanosoma evansi, T. vivax and T. congolense in camels (Camelus dromedarius) of KSA
Source: BMC Vet Res. 2022 Jan 18;18:45. doi: 10.1186/s12917-022-03148-0 (PMC8764778; doi:10.1186/s12917-022-03148-0)

File: ILO-FP-D3=_H05--RSG-29-21-Supp.ab1

Geospiza
www.geospiza.com

Sample Name: ILO-FP-D3
Mobility: KB_3500_POP7_BDTv3.mob
Spacing: 10.4989
Comment: n/a

Signal Strengths: A = 1811, C = 2229, G = 1438, T = 1390
Lane/Cap#: 8
Matrix: n/a
Direction: Native

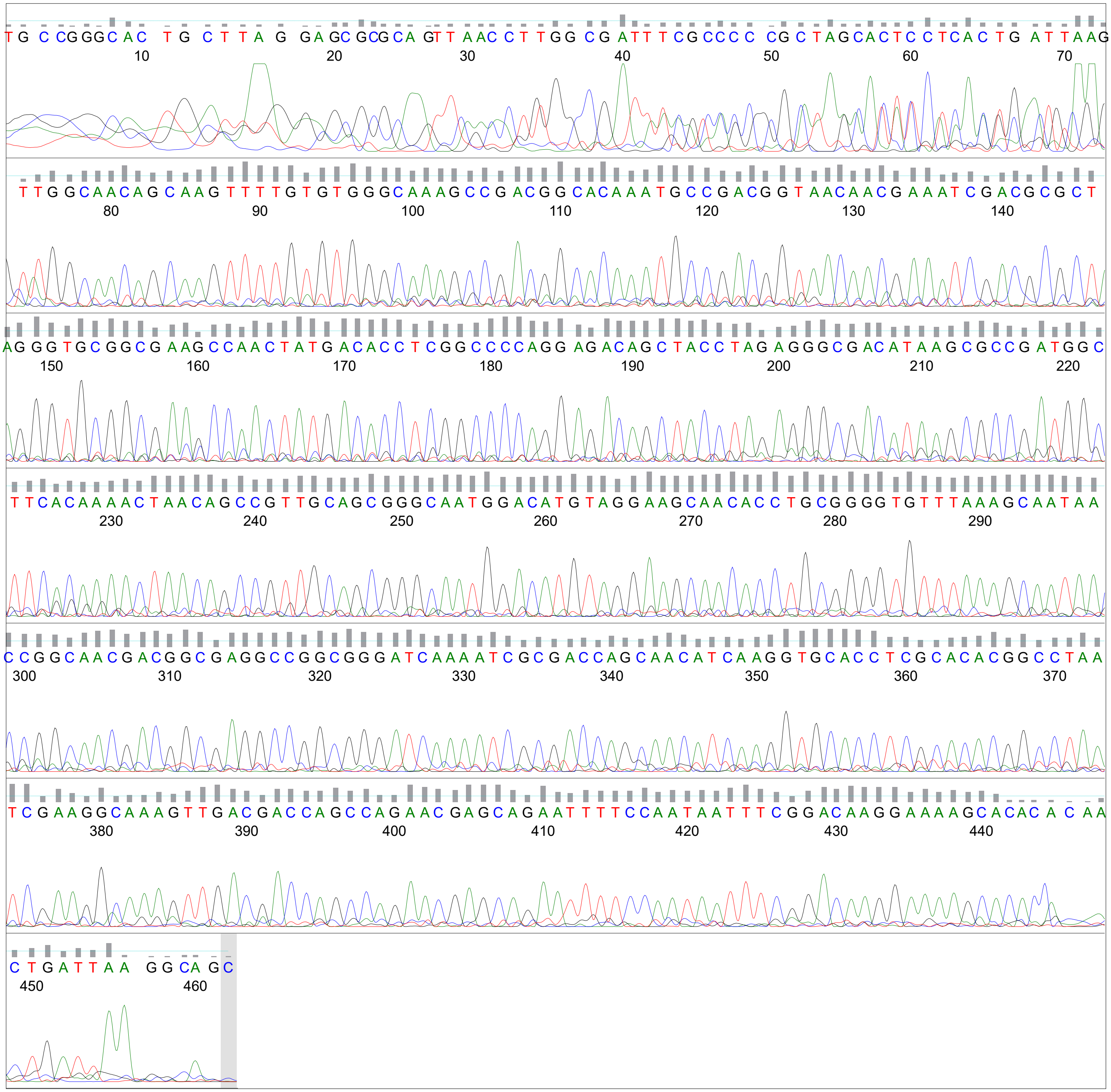

Supplement: Supplementary file 1 — Additional file 1. [file 12917_2022_3148_MOESM1_ESM.pdf]
