# Supplementary material for: Molecular characterization of Trypanosoma evansi, T. vivax and T. congolense in camels (Camelus dromedarius) of KSA
Source: BMC Vet Res. 2022 Jan 18;18:45. doi: 10.1186/s12917-022-03148-0 (PMC8764778; doi:10.1186/s12917-022-03148-0)

File: ILO-FP-D4=_A06--RSG-29-21-Supp.ab1

Geospiza
www.geospiza.com

Sample Name: ILO-FP-D4	Signal Strengths: A = 1228, C = 1497, G = 1025, T = 935
Mobility: KB_3500_POP7_BDTv3.mob	Lane/Cap#: 1
Spacing: 10.4924	Matrix: n/a
Comment: n/a	Direction: Native

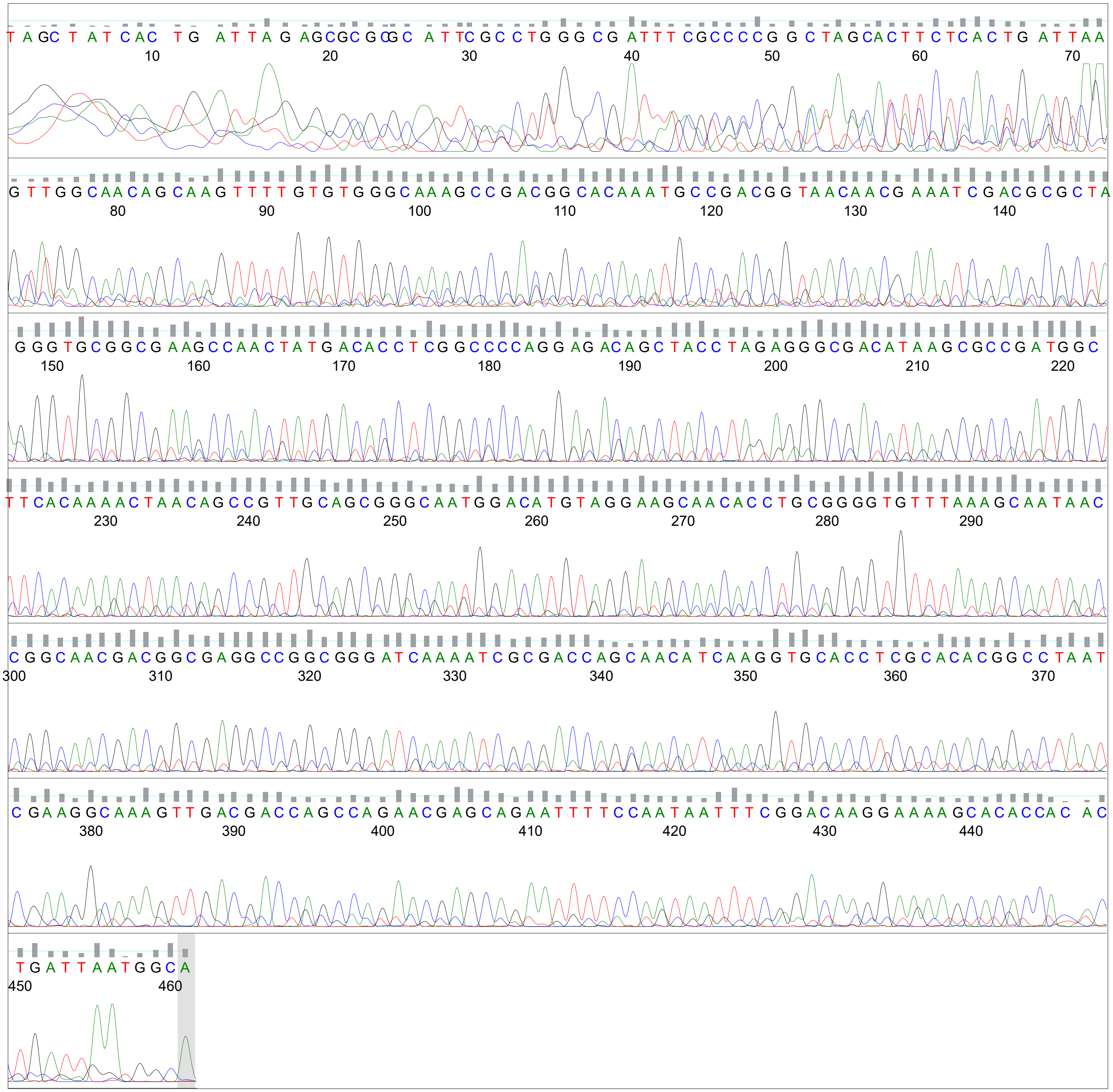

Supplement: Supplementary file 2 — Additional file 2. [file 12917_2022_3148_MOESM2_ESM.pdf]
